# Supplementary material for: Prevalence and factors associated with food insecurity across an entire campus population
Source: PLoS One. 2020 Aug 31;15(8):e0237637. doi: 10.1371/journal.pone.0237637 (PMC7458338; doi:10.1371/journal.pone.0237637)
Supplement: S1 Table — (DOCX) [file pone.0237637.s001.docx]

Supplementary Table 1. Margin of error calculations, total response number and campus population of a given demographic group at the time of the survey.

|  | Spring 2017 | | | Fall 2017 | | |
| --- | --- | --- | --- | --- | --- | --- |
| Demographic Group | Margin of Error | Response number | Campus Population | Margin of Error | Response number | Campus Population |
| Undergraduate | 4.07% | 547 | 9786 | 3.81% | 624 | 10513 |
| Grad | 9.35% | 102 | 1406 | 9.53% | 99 | 1517 |
| Med | 13.24% | 49 | 457 | 16.73% | 32 | 459 |
| Faculty | 9.02% | 110 | 1600 | 9.20% | 106 | 1600 |
| Staff | 6.16% | 229 | 2373 | 5.71% | 262 | 2373 |
| Overall | 2.94% | 1037 | 15622 | 2.82% | 1123 | 16462 |
