# Supplementary material for: Prevalence and factors associated with food insecurity across an entire campus population
Source: PLoS One. 2020 Aug 31;15(8):e0237637. doi: 10.1371/journal.pone.0237637 (PMC7458338; doi:10.1371/journal.pone.0237637)
Supplement: S2 Table. a. ANOVA results of food insecurity outcomes by demographic group with Bonferroni multiple comparison tests- Spring 2017. b. ANOVA results of food insecurity outcomes by demographic group with Bonferroni multiple comparison tests- Fall 2017 — (DOCX) [file pone.0237637.s002.docx]

Supplementary Table 2a. ANOVA results of food insecurity outcomes by demographic group with Bonferroni multiple comparison tests- Spring 2017

| Row Mean-Column Mean | Undergraduate | Graduate | Medical | Faculty |
| --- | --- | --- | --- | --- |
| Graduate | -0.073 (0.840) |  |  |  |
| Medical | -0.096 (0.995) | -0.0230 (1.000) |  |  |
| Faculty | -0.241 (0.000) | -0.168 (0.017) | -0.145 (0.298) |  |
| Staff | -0.123 (0.001) | -0.051 (1.000) | -0.028 (1.000) | 0.117 (0.094) |

Supplementary Table 2b. ANOVA results of food insecurity outcomes by demographic group with Bonferroni multiple comparison tests- Fall 2017

| Row Mean-Column Mean | Undergraduate | Graduate | Medical | Faculty |
| --- | --- | --- | --- | --- |
| Graduate | 0.033 (1.000) |  |  |  |
| Medical | 0.025 (1.000) | -0.008 (1.000) |  |  |
| Faculty | -0.127 (0.008) | -0.161 (0.014) | -0.152 (0.383) |  |
| Staff | -0.064 (0.156) | -0.099 (0.206) | -0.090 (1.000) | 0.062 (1.000) |
