# Supplementary material for: Prevalence and factors associated with food insecurity across an entire campus population
Source: PLoS One. 2020 Aug 31;15(8):e0237637. doi: 10.1371/journal.pone.0237637 (PMC7458338; doi:10.1371/journal.pone.0237637)
Supplement: S3 Table. a. ANOVA results of food insecurity outcomes by school year with Bonferroni multiple comparison tests- Spring 2017. b. ANOVA results of food insecurity outcomes by school year with Bonferroni multiple comparison tests- Fall 2017 — (DOCX) [file pone.0237637.s003.docx]

Supplementary Table 3a. ANOVA results of food insecurity outcomes by school year with Bonferroni multiple comparison tests- Spring 2017

| Row Mean-Column Mean | 1st year | 2nd year | 3rd year |
| --- | --- | --- | --- |
| 2nd year | 0.082 (1.000) |  |  |
| 3rd year | 0.165 (0.025) | 0.083 (0.888) |  |
| 4th-6th year | 0.148 (0.075) | 0.067 (1.00) | -0.016 (1.000) |

Supplementary Table 3b. ANOVA results of food insecurity outcomes by school year with Bonferroni multiple comparison tests- Fall 2017

| Row Mean-Column Mean | 1st year | 2nd year | 3rd year |
| --- | --- | --- | --- |
| 2nd year | 0.014 (1.000) |  |  |
| 3rd year | 0.096 (0.726) | 0.082 (1.000) |  |
| 4th-6th year | 0.144 (0.036) | 0.130 (0.114) | -0.048 (1.000) |
